# Wall Teichoic Acids of *Staphylococcus aureus* Limit Recognition by the Drosophila Peptidoglycan Recognition Protein-SA to Promote Pathogenicity

**DOI:** 10.1371/journal.ppat.1002421

**Published:** 2011-12-01

**Authors:** Magda L. Atilano, James Yates, Marcus Glittenberg, Sergio R. Filipe, Petros Ligoxygakis

**Affiliations:** 1 Laboratory of Bacterial Cell Surfaces and Pathogenesis, Instituto de Tecnologia Química e Biológica/Universidade Nova de Lisboa, Oeiras, Portugal; 2 Genes and Development Laboratory, Department of Biochemistry, University of Oxford, Oxford, United Kingdom; Stanford University, United States of America

## Abstract

The cell wall of Gram-positive bacteria is a complex network of surface proteins, capsular polysaccharides and wall teichoic acids (WTA) covalently linked to Peptidoglycan (PG). The absence of WTA has been associated with a reduced pathogenicity of *Staphylococcus aureus (S. aureus)*. Here, we assessed whether this was due to increased detection of PG, an important target of innate immune receptors. Antibiotic-mediated or genetic inhibition of WTA production in *S. aureus* led to increased binding of the non-lytic PG Recognition Protein-SA (PGRP-SA), and this was associated with a reduction in host susceptibility to infection. Moreover, PGRP-SD, another innate sensor required to control wild type *S. aureus* infection, became redundant. Our data imply that by using WTA to limit access of innate immune receptors to PG, under-detected bacteria are able to establish an infection and ultimately overwhelm the host. We propose that different PGRPs work in concert to counter this strategy.

## Author Summary

Gram-positive bacteria such as the opportunistic pathogen *Staphylococcus aureus* have their cell wall exposed to the environment found within a host. Following an infection these bacteria need to find ways to evade or reduce recognition by the host in order to survive and potentially proliferate. The cell wall of Gram-positive bacterium consists of an intricate network of glycan chains cross-linked by short peptides called peptidoglycan (PG; a major target for host recognition in a variety of animals) covalently linked to surface proteins and glycopolymers including Wall Teichoic Acids (WTA). It has been proposed that lack of WTA reduce the pathogenicity of *S. aureus*. We asked whether this was due to better recognition of PG. We found that both bacterial recognition and survival of fruit flies (our model host) infected with bacteria lacking WTA was markedly increased compared to those infected with wild type *S. aureus*. This result was quantifiable: a reduction in the amount of WTA resulted in greater binding by host receptors and a higher host survival. We propose that the presence of WTA limit access to PG and therefore reduce the recognition ability of the host. Bacteria are thus able to increase in numbers and eventually overwhelm the host.

## Introduction

The complex cell surface of bacteria has been directly or indirectly associated with different strategies that bacterial pathogens use to interact with the host. These include acquisition of specific adhesion factors, formation of biofilms, adaptation to an intracellular environment, production of a protective capsular polysaccharide or evasion of innate immune defences (e.g. lysozyme) [1]. The host counters these strategies by targeting conserved molecules (pathogen associated molecular patterns or PAMPs), unique in bacteria, that are either present at the bacterial surface or are released by bacteria as they attempt to establish infection. Bacterial PAMPs include Peptidoglycan (PG), a heterogeneous polymer of glycan chains cross-linked by short peptides of variable length and amino acid composition [2]. Although PG recognition is essential to trigger an inflammatory response, this macromolecule may not be easily accessible for recognition at the surface of bacteria.

In Gram-positive bacteria, PG is buried within a complex cell surface consisting of different molecules [3]–[5]. Such molecules include surface proteins, covalently linked or tightly associated with PG, capsular polysaccharides, usually required for the ability of different bacteria to cause disease [6] and wall teichoic acids (WTA), phosphate-rich glycopolymers involved in the resistance of bacteria to environmental stress and regulation of bacterial division [7]. It is not clear therefore, how the host would be able to sense bacterial PG buried within such complex structures. One hypothesis is that the innate immune system recognises soluble PG fragments that are released from the bacterial cell surface through the activity of enzymes produced by bacteria (such as autolysins) or by the host (such as lysozyme) [2], [8]. However, certain bacteria have the ability to modify their PG, turning it more resistant to the action of such enzymes [9], thus preventing the release of small soluble fragments capable of triggering an innate immune response in the host. This may be the case for *Listeria monocytogenes* that has the ability to de-*N*-acetylate its PG allowing them to survive the action of lysozyme and evade the host innate immune system [10]. Another hypothesis is that the components of the host innate immune system are able to bind directly to PG present within the bacterial cell surface. As discussed earlier, PG is decorated with a variety of large molecules that may sterically block access of host receptors to the underlying PG. In Gram-positive bacteria, cell wall glycopolymers, including WTA may play this role [1]. The role of WTA protecting the PG from recognition would have important implications regarding the onset of infection by major human pathogens such as *Staphylococcus aureus* (*S. aureus*) [1]. Recently, it has been shown that different components, present at the cell wall of *S. aureus* bacteria, may determine the survival of infected *Drosophila*. Specifically, *S. aureus* strains impaired in the expression of enzymes involved with the metabolism of cell wall components were unable to kill flies [11]. Moreover, it has been proposed that D-alanylation of the WTA produced by *S. aureus* may inhibit the recognition of PG by host receptors. This inhibitory effect was observed *in vitro* not only when WTA was covalently attached to polymeric PG but, surprisingly, also when WTA was covalently attached to monomeric PG [12].

The fruit fly *Drosophila melanogaster* recognises Gram-positive bacteria by either direct binding to PG or its smallest components [13]. Based on *in vitro* data [14] and infection studies of mutants [14], [15], the current working hypothesis is that a flexible system of pattern recognition receptors (PRRs) can be deployed by the host immune system to detect Lysine-type PG from different Gram-positive bacterial pathogens. Two Peptidoglycan Recognition Proteins (PGRPs), namely PGRP-SA and PGRP-SD are major components of this system [15], [16]. Depending on the bacterium, each, or both of these PGRPs – along with Gram-Negative Binding Protein1 (GNBP1) [17] – interacts with PG and activate a downstream proteolytic cascade, which culminates in Toll receptor signalling. The signal reaches the cytoplasmic NF-κB/I-κB complex via a receptor/adaptor complex comprising dMyD88, Tube and the IRAK homologue Pelle. At that point the I-κB homologue Cactus is phosphorylated and targeted for degradation while the NF-κB homologue Dif is free to enter the nucleus of host cells and regulate target genes [18]. Prominent among these genes, is a group of potent antimicrobial peptides (AMPs), which are synthesised by the fat body and secreted into the haemolymph. An AMP frequently used as a read-out for the Toll pathway is *Drosomycin* (*Drs*). AMPs and local melanization, along with the phagocytic activity of haemocytes constitute respectively the humoral and cellular arm of the fruit fly response to infection [18].

Here, we report for the first time that Drosophila PGRP-SA, a non-lytic PGRP was able to bind intact live bacteria *in vivo*. Access to PG was limited by the presence of WTA: binding of PGRP-SA to various live Gram-positive bacteria was minimal, but binding to purified PG, stripped of covalent modifications (including WTA) was far greater. Through inhibiting WTA synthesis, either by the addition of an antibiotic or genetically, we were able to potentiate detection of these bacteria by PGRP-SA. For *S*. *aureus*, this correlated with a reduced ability of the bacteria to proliferate within the host, and a reduced susceptibility of the host to infection in a PGRP-SA/GNBP1 dependent manner. We also observed that PGRP-SD, essential for sensing wild type *S*. *aureus*, became redundant as WTA levels were reduced. Overall, our results suggest that WTA may be part of a general mechanism used by Gram-positive bacteria, which limits the access of innate receptors to PG, thereby enabling bacteria to evade detection and establish infection.

## Results

To address the question of whether Gram-positive bacteria counter host recognition by limiting access of innate sensors to PG, we constructed a fluorescent derivative of the fruit fly Lys-type PG receptor, PGRP-SA (mCherry-PGRP-SA). This construct and an untagged version (rPGRP-SA) were expressed in *Escherichia coli* and the resulting proteins were purified. As shown in the supplementary material (Figure S1A), injection of mCherry-PGRP-SA, or rPGRP-SA, into PGRP-SA deficient flies restored *Drs-GFP* production induced by infection with *Micrococcus luteus* (*M. luteus*). Endogenous *Drs* expression was also restored as confirmed by qPCR (Figure S1B). These observations were consistent with our previous results when using a recombinant PGRP-SA expressed in the lepidopteran cell line Sf9 [19]. Taken together, these results showed that the fluorescently tagged PGRP-SA and the untagged versions are functional and capable of restoring an innate immune response in PGRP-SA deficient flies.

**Figure 1:**
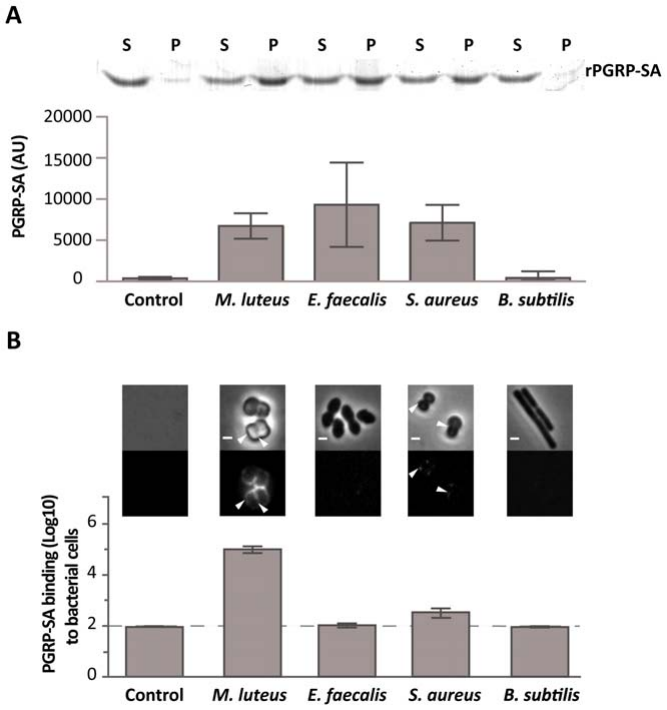
Differential binding of PGRP-SA to the surface of live Gram-positive bacteria. (**A**) PGRP-SA and PG co-precipitation assay. Lys-type PG from *M. luteus*, *E. faecalis*, *S. aureus*, and DAP-type PG from *B. subtilis* (this acts as a negative substrate control for PGRP-SA binding, which recognizes Lys-type PG), was incubated with rPGRP-SA for 30 minutes. Unbound rPGRP-SA remained in the supernatant fraction upon centrifugation (S). rPGRP-SA bound to the insoluble PG was co-precipitated and found in the pellet fraction (P). Quantified data (performed using ImageJ software) was plotted as mean values with 95% confidence limits: very little co-precipitation of rPGRP-SA occurred in the absence of PG (labelled Control) or in the presence of *B. subtilis* DAP-type PG; however, PGRP-SA was co-precipitated similarly (One-way ANOVA, *P*>0.05) and at higher levels with the PG from *M. luteus*, *E. faecalis*, or *S. aureus*. The data shown (mean with 95% confidence intervals) was obtained from 4 independent co-precipitation experiments. (**B**) mCherry-PGRP-SA was incubated with bacteria cells harvested in exponential phase, washed with PBS and visualized using fluorescence microscopy. Grey panels are phase-contrast images of bacterial cells (white scale bar represents 1 µm), and black panels mCherry-PGRP-SA binding: white arrowheads highlight binding to the lateral cell surface or the region of cell division. The total fluorescence of mCherry-PGRP-SA bound to a bacterium (covering all lateral and cell division regions, and including background) was quantified for each species (*n* = 50), and represented as the median (with 25% and 75% inter-quartile range). Dashed-line indicates the level of the background signal, control. Kruskal-Wallis analysis with Dunn's multiple comparison post-test did not reveal significant differences (*P*>0.05) between mCherry-PGRP-SA binding to *E. faecalis* and *B. subtilis*, which were indistinguishable from the control. However, the protein bound more to *S. aureus* and *M. luteus* relative to the control, with the latter exhibiting highest binding (*P*<0.05 in all cases).

Initially, we used rPGRP-SA and mCherry-PGRP-SA in co-precipitation experiments in order to study binding to PG from different Gram-positive bacteria. Both bound with similar affinity to PG purified from *M. luteus*, *Enterococcus faecalis* (*E. faecalis*), and *S. aureus* (data not shown and Figure 1A, respectively). For details of PG composition of these bacteria see Figure S2. Importantly, this indicated that the mCherry-tag appeared not to interfere with PGRP-SA binding, and thus, demonstrated that both proteins were able to bind Lys-type PG of different composition. We therefore assessed *in vitro*, the binding of mCherry-PGRP-SA to the surface of live bacteria harvested during exponential growth phase. Notably, the binding of the recombinant protein to live bacteria exhibited a range of different affinities in contrast to their respective purified PG. Binding to live *E. faecalis* and *S. aureu*s was significantly reduced, when compared to binding to *M. luteus* (Figure 1B). However, the binding levels of PGRP-SA to the purified PG from these bacteria were similar (Figure 1A). We also noticed that while mCherry-PGRP-SA was capable of binding the entire surface of *M*. *luteus* cells, it bound at specific sites at the surface of *S. aureus* cells, similar to what has been described recently for mammalian bactericidal PGRPs [20]. These results suggested that although the three types of bacterial PG were similarly recognized by PGRP-SA, the presence of other components found at the surface of live bacteria might have prevented PGRP-SA from finding its PG ligand.

**Figure 2:**
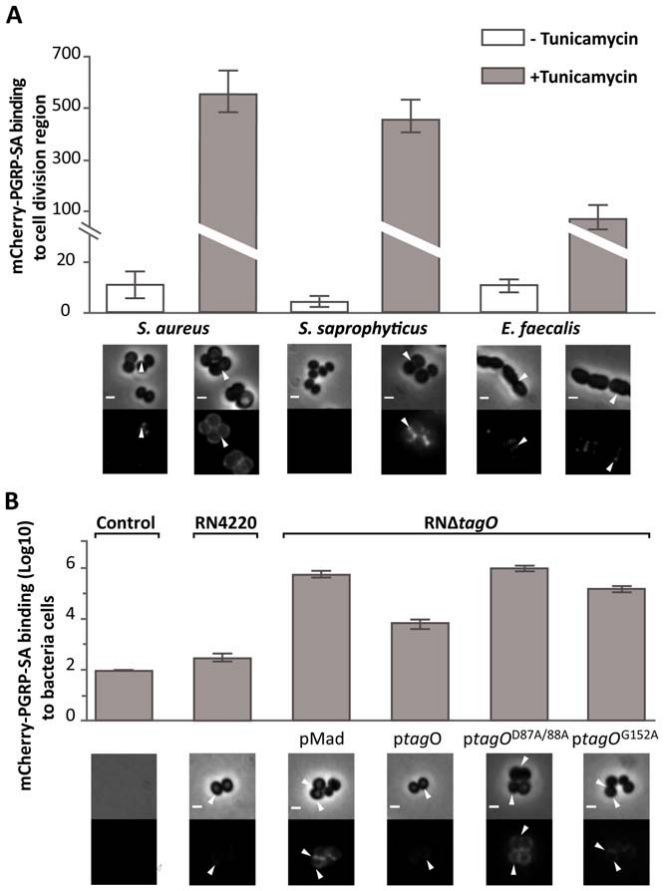
WTA reduce PGRP-SA binding at the bacterial cell surface. Grey panels are phase-contrast images of bacterial cells (white scale bar represents 1 µm), and black panels mCherry-PGRP-SA binding; white arrowheads highlight binding to the lateral cell surface or region of cell division. The binding of mCherry-PGRP-SA to individual bacterial cells (*n* = 50) was quantified, and represented as the median (with 25% and 75% inter-quartile range). (A) mCherry-PGRP-SA binding to Gram-positive bacteria grown with or without tunicamycin, an inhibitor of WTA synthesis. mCherry-PGRP-SA binding to the cell division region, rather than total binding, was measured because binding at the former was consistently enhanced for all treated bacteria species. Mann-Whitney U tests were used to compare differences for treated and untreated between each type of bacteria (*P*<0.05 in all cases). (B) RNΔ*tagO* mutant background rescued with variants of the *tagO* gene – expressed from a replicative pMAD vector – produce varying levels of WTA, given as a% relative to the wild type RN4220: pMAD vector (0%), p*tagO* (90%), ptagOD87A/D88A (0%), ptagOG152A (22%). Total binding of mCherry-PGRP-SA to the surface of live bacteria increases as the levels of WTA are reduced. Kruskal-Wallis analysis followed by Dunn's multiple comparison post-test, revealed significant differences for all comparisons (*P*<0.05) except for that between PGRP-SA binding to pMAD and ptagOD87A/D88A.

The cell surface of a Gram-positive bacterium is a complex structure consisting of a thick layer of PG, surface proteins and glycopolymers such as capsular polysaccharides and WTA. As previous studies had shown that certain PG-binding proteins, such as bacterial autolysins, have a higher affinity for the surface of bacterial strains lacking WTA [21]–[23], it was decided to investigate whether presence of WTA could be preventing PGRP-SA from binding to the surface of live bacteria. Further support for the choice of WTA came from the fact that different Gram-positive bacteria can produce WTA with a variable composition [24]–[26]. *M. luteus*, for which mCherry-PGRP-SA displayed the highest affinity, does not produce WTA [24], [27] **,** (Figure S2C). To test whether WTA mediated the differential binding of PGRP-SA, we cultured bacteria in the presence of tunicamycin, thereby inhibiting their ability to synthesize WTA. At lower, sub-inhibitory concentrations as those used in this study, tunicamycin specifically inhibits TagO [28]: a glycosyltransferase that specifically localizes to the division septum of *S.* aureus [29] and is required for the initial step of WTA biosynthesis, namely, the transfer of GlcNAc to the C55-P lipid anchor bactoprenol. We observed higher levels of mCherry-PGRP-SA binding to the newly synthesized cell material, located at the division septum, when Gram-positive bacteria cells were treated with tunicamycin (Figure 2A). *S. aureus* and *S*. *saprophyticus* exhibited a similar increase in binding, 63× and 84× respectively, whilst *E*. *faecalis* binding increased 8×. It should be noted that the effect of tunicamycin in these bacteria was not the same. While addition of the antibiotic resulted in binding of mCherry-PGRP-SA to the entire cell surface of *S*. *aureus*, binding was observed predominantly at the division septum in *S*. *saprophyticus* and exclusively at this region in *E*. *faecalis*. We attribute these differences to how and where the new cell wall synthesis occurs in these bacteria. Nevertheless, the results described above suggested that WTA in different bacteria might protect PG from exposure to host receptors.

To confirm that WTA were indeed required to reduce access of PGRP-SA at the cell surface, we quantified the binding of mCherry-PGRP-SA to *S*. *aureus* mutants that produced varying amounts of WTA due to mutations in the *tagO* gene [29]. We chose *S*. *aureus* because it is a major human pathogen with a well-characterised WTA synthetic pathway [30], [31]. A complete absence of WTA, which occurs when *tagO* is entirely deleted (RNΔ*tagO* pMAD), or when two highly conserved residues have been mutated (RNΔ*tagO* p*tagO* ^D87A/D88A^), resulted in equivalently enhanced levels of mCherry-PGRP-SA binding, when compared to the wild type strain (∼2×10^3^ and ∼3.3×10^3^-fold respectively, Figure 2B). To verify that the observed result was indeed due to the loss of WTA, we expressed wild type *tagO* in the RNΔ*tagO* background (RNΔ*tagO* p*tagO*): this rescued the loss of WTA (WTA levels restored to 90% of wild type levels) [29], and reduced mCherry-PGRP-SA binding to levels close to those observed for the wild type strain (Figure 2B). A *tagO* mutant that could only support production of a reduced amount of WTA (RNΔ*tagO* p*tagO* ^G152A^; 24% levels of WTA compared to wild type) exhibited an intermediate level of mCherry-PGRP-SA binding relative to all strains (6×10^2^-fold increase relative to the wild type strain, Figure 2B). Overall, our data indicated that WTA found in the cell wall of different live Gram-positive bacteria restricted PGRP-SA from binding their PG, and in *S. aureus* this occurs in a dose dependent manner.

**Figure 3:**
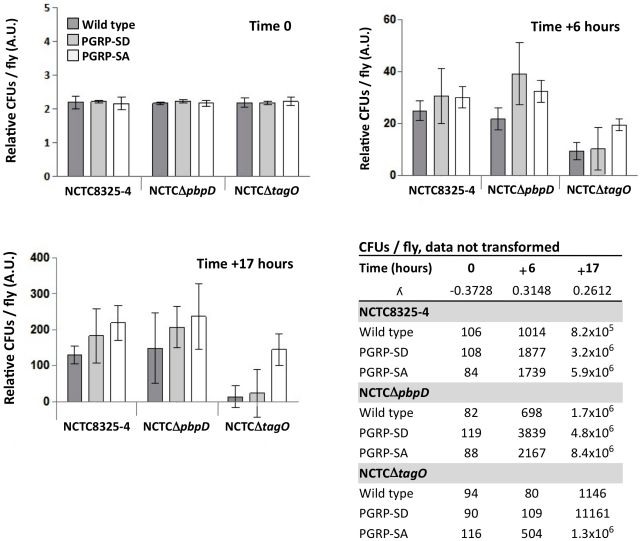
PGRP-SA is fundamental for controlling bacterial numbers in flies infected with a *S. aureus* mutant that lacks WTA. Wild type flies, and those lacking PGRP-SD or PGRP-SA, were infected with different *S. aureus strains*: NCTC8325-4 is the wild type; NCTCΔ*pbpD* is a mutant that produces WTA but has a PG similar to NCTCΔ*tagO*, both exhibiting reduced cross-linking; NCTCΔ*tagO* lacks WTA. The table gives the mean CFUs per fly (from 3 independent experiments). For each time point, the CFUs per fly data set was transformed via a Box-Cox transformation (which returns a λ number, where data-point  =  data-point^λ^ – 1/λ) and represented as means with 95% confidence intervals. Flies were inoculated with a low (∼100 CFUs per fly) and comparable number of bacteria (Time 0; Two-way ANOVA did not reveal significant differences, *P*>0.05), and CFUs per fly were determined at 6 and 17 hours post-infection. In contrast to NCTC8325-4 and NCTCΔ*pbpD*, the number of NCTCΔ*tagO* bacteria did not significantly increase in the wild type or PGRP-SD mutant background during the period of infection (Table); however, in the PGRP-SA mutant the number of bacteria increased significantly for all strains (*P*<0.05, Repeated Measures One-way ANOVA). Two-way ANOVA of the CFUs data at Time +17 hours revealed a significant interaction (*P*<0.05) between the bacteria and fly strains, which was due to the large increase of NCTCΔ*tagO* CFUs in the PGRP-SA mutant, whilst differences in CFUs were similar for NCTC8325-4 and NCTCΔ*pbpD*. One-way ANOVA and 95% Tukey's HSD intervals were used to look for factor differences at this time. For each fly background NCTC8325-4 and NCTCΔ*pbpD* CFUs were equivalent (*P*>0.05). NCTCΔ*tagO* CFUs in the wild type and PGRP-SD backgrounds were similar (*P*>0.05), but separated from all other values (*P*<0.05). In the PGRP-SA mutant, NCTCΔ*tagO* CFUs reached levels seen with the other bacteria in wild type and PGRP-SD flies. The negative error bars for the NCTCΔ*tagO* infection occur because of large variation of the biological repeats. This is consistent with the fact that NCTCΔ*tagO* occasionally causes a lethal infection in both the wild type and PGRP-SD backgrounds.

We next wanted to examine whether increased PGRP-SA binding – due to a lack of WTA – affected the ability of bacteria to survive in an *in vivo* system. We chose *D. melanogaster* because it is a well-established model for dissecting pattern recognition in innate immunity [18]. We know for example that *in vitro*, three PRRs – PGRP-SD/PGRP-SA/GNBP1 – form a ternary complex for binding to the PG of *S*. *aureus* [14]. As a first approach wild type and mutant *S. aureus* strains were injected into wild type flies and also into flies defective for PGRP-SD or PGRP-SA. We then determined the number of CFUs 6 and 17 hours post-infection; the latter time point being when the first flies succumb to infection (Figure 3 and S5). All flies were inoculated with low and statistically identical numbers of bacteria (∼10^2^ CFUs per fly; Figure 3, Time 0). Our rationale was to induce infections that were comparable and that could evolve over time. For example, flies generally succumb to bacterial infection when their numbers increase beyond 10^6^ CFUs per fly [18], [32], and therefore, high initial loads (e.g. 10^4^–10^5^ CFUs per fly) may overwhelm the host and consequently may not be informative regarding the course of an infection. We observed that wild type *S*. *aureus* (NCTC8325-4) CFUs increased in all fly backgrounds over the period of infection to numbers that were statistically separable, with PGRP-SA deficient flies carrying the heaviest load (Figure 3). In contrast, the numbers of the *S*. *aureus* mutant, which lacked WTA (NCTCΔ*tagO*) [29], did not significantly increase in the wild type or PGRP-SD mutant background. However, the number of NCTCΔ*tagO* bacteria in the PGRP-SA mutant was significantly higher at both the 6 and 17 hours time points (Figure 3). Two-way ANOVA revealed a significant interaction between the bacteria and fly strains, which was due to the large increase of NCTCΔ*tagO* bacteria in the PGRP-SA mutant. Together, these data indicated that WTA were fundamental for *S*. *aureus* to counter recognition by PGRP-SA, and consequently, the bacteria were able to increase their number during the initial course of infection.

We have previously observed that PG produced by NCTCΔ*tagO* bacteria has reduced levels of cross-linking relative to the wild type strain [29]. To evaluate whether this contributed to the inability of NCTCΔ*tagO* bacteria to increase their number in wild type or PGRP-SD mutant flies, we assessed mCherry-PGRP-SA binding to NCTCΔ*pbpD* and determined CFUs at 6 and 17 hours. NCTCΔ*pbpD* is a derivative of NCTC8325-4 in which *pbpD* (the gene encoding to penicillin binding protein 4, PBP4) has been deleted. Deletion of *pbpD* results in a strain that produces PG with a similar level of cross-linking to that found in NCTCΔ*tagO* [29], but which still produces WTA. The inability of NCTCΔ*pbpD* and NCTCΔ*tagO* to produce a highly crosslinked PG did not interfere with bacteria growth in culture, as its duplication time at 30°C was very similar to the parental NCTC8325-4 strain (Figure S3B). In both experiments, NCTCΔ*pbpD* behaved as the wild type bacteria. Firstly, binding mcherry-PGRP-SA similarly (Figure S3C) and secondly, for each fly background attaining numbers that were statistically inseparable from those for NCTC8325-4 (Figure 3, Time +17 hours).

**Figure 4:**
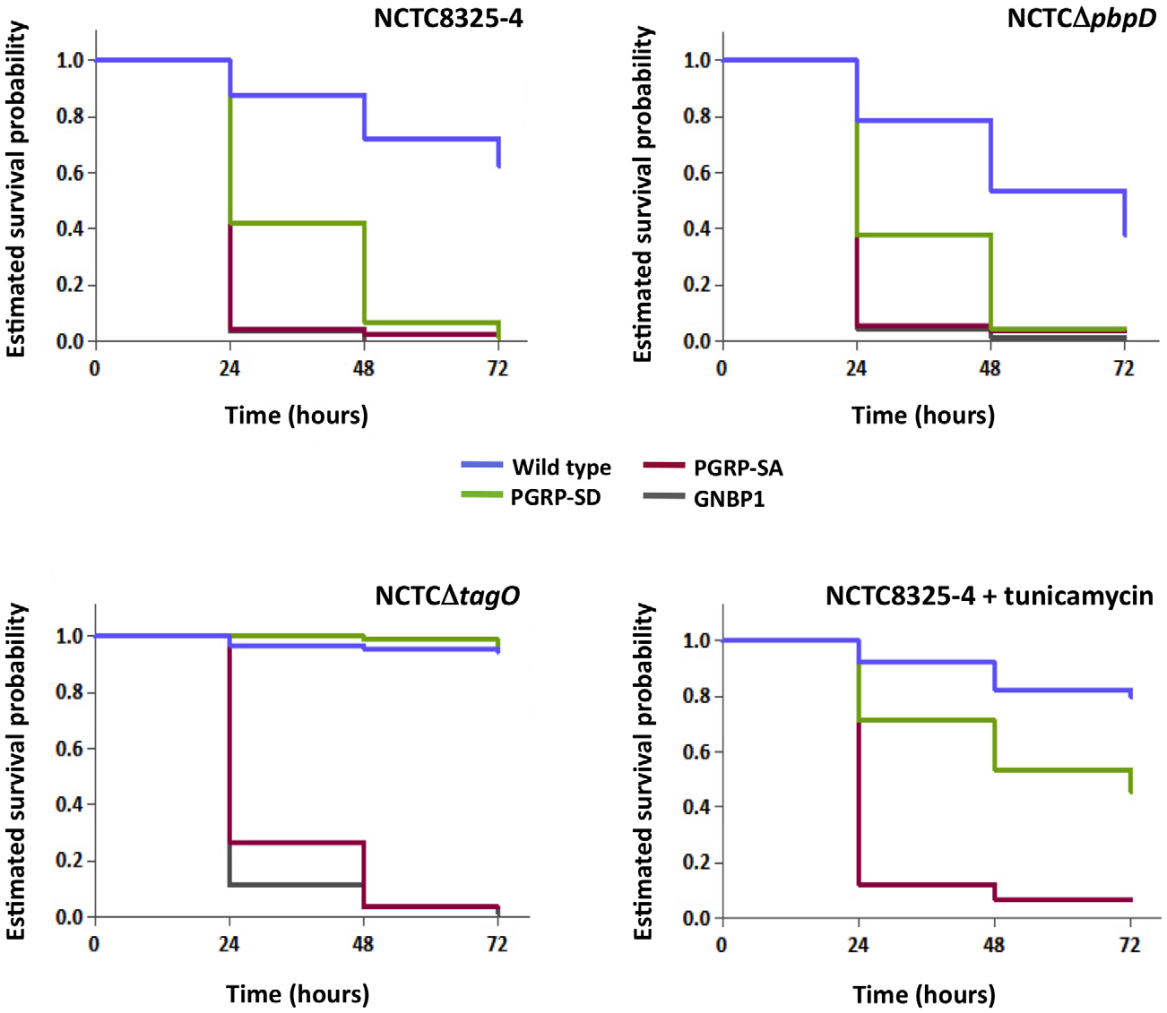
PGRP-SA and not PGRP-SD is required to control infection by *S. aureus* mutant lacking WTA. Flies assayed for survival were injected concurrently with those for determining CFUs. The survival of infected flies (*n* = 90) was monitored at 24-hour intervals for three days, and estimates of survival plotted (for clarity, 95% confidence intervals have been omitted). For each fly background – except wild type – survival curves were statistically inseparable for flies infected with NCTC8325-4 or NCTCΔ*pbpD* (log-rank test, *P*>0.05). PGRP-SD, PGRP-SA and GNBP1 mutant flies succumbed strongly to infection by 72 hours, whereas wild type survived up to ∼60%. In contrast, wild type and PGRP-SD mutant flies were barely susceptible to infection with NCTCΔ*tagO*, however, PGRP-SA and GNBP1 flies succumbed strongly; a similar survival trend was seen when flies were infected with tunicamycin-treated NCTC8325-4 (GNBP1 mutant flies were not infected for this experiment).

To assess whether the developing trend in bacterial numbers at 17 hours post-infection resolved into differences in how flies survive, we monitored the number of flies alive at 24 hour intervals over 3 days. In addition, we infected GNBP1 mutant flies, because GNBP1 has been postulated to work as part of a complex with PGRP-SA [14], [17]. Survival curves for a particular fly background when infected with either NCTC8325-4 or NCTCΔ*pbpD* were statistically inseparable, except for those obtained for the wild type background, where flies succumbed more to NCTCΔ*pbpD* (Figure 4; 62% and 38% survival at 72 hours post-infection, respectively). Nearly all PGRP-SA and GNBP1 mutant flies had died by 24 hours, whereas ∼40% of PGRP-SD mutant flies survived beyond this time point, succumbing to infection around 48 hours (∼5% of flies surviving). In contrast, ∼95% of wild type and PGRP-SD mutant flies survived the NCTCΔ*tagO* infection up to 72 hours (furthermore, taking CFUs at this time-point revealed that NCTCΔ*tagO* had been eliminated from these flies, 0 CFUs per fly). The majority of PGPR-SA and GNBP1 flies had succumbed to infection by 48 hours (3% of flies surviving). A similar trend in survival outcome was observed with NCTC8325-4 after treatment with tunicamycin (Figure 4). These data confirmed that WTA were indeed required to counter host immunity, because without them, infection could be controlled in a PGRP-SA/GNBP1 dependent manner. Differences in CFUs were apparent 6 hours post-infection suggesting that recognition and reduction of propagation or killing of bacteria, occurs rapidly following infection. Interestingly, these results also showed that a requirement for PGRP-SD was bypassed when WTA are removed and PGRP-SA has far greater access to PG.

**Figure 5:**
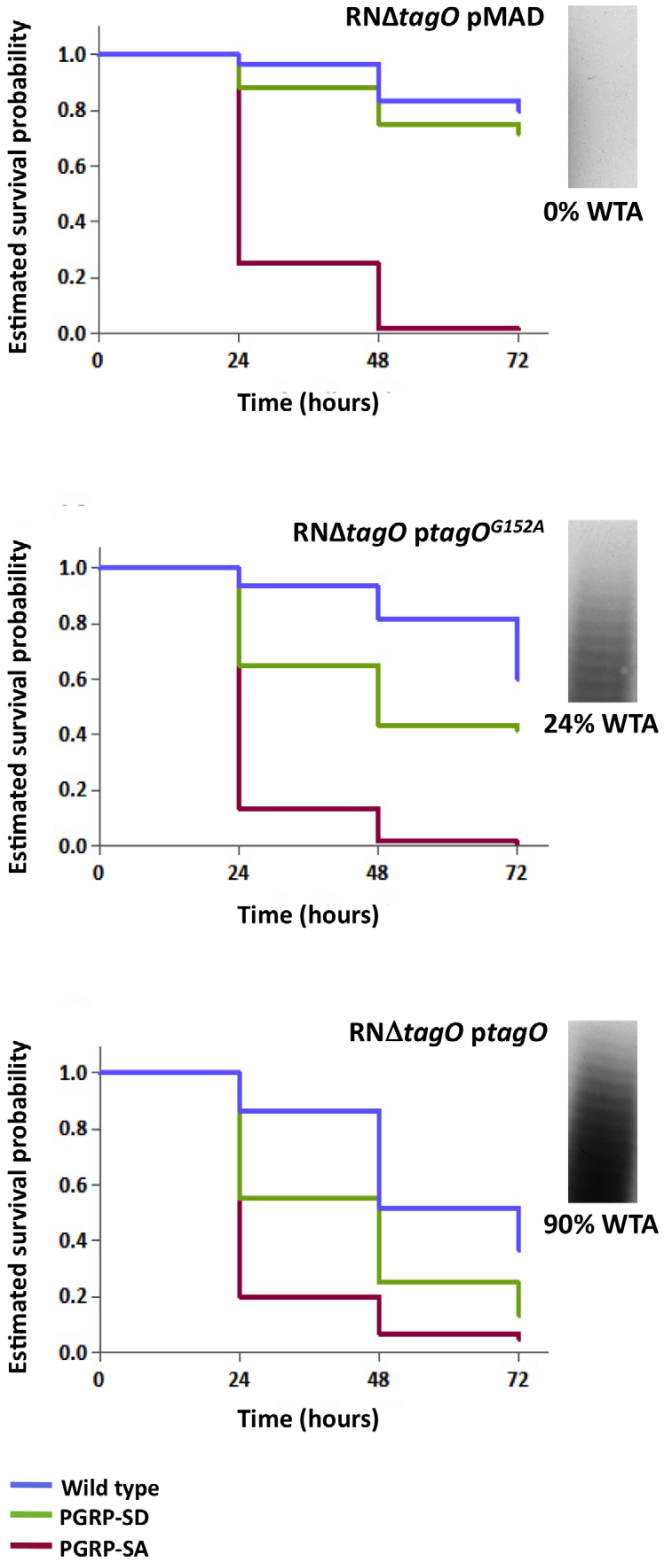
The levels of WTA modulate the requirement for PRRs. Survival of infected flies (*n* = 60) was monitored at 24 hours intervals for three days, and estimates of survival constructed from the raw data. Flies were infected with *S. aureus* mutants that produce different levels of WTA (percentage of WTA produced by each strain was quantified as the signal intensity of bands of WTA in the native gels, and it was normalized against the corresponding value for the wild type – considered as 100%): RNΔ*tagO* pMAD lacks WTA; RNΔ*tagO* p*tagO* produces 90% WTA relative to the parental RN4220; and RNΔ*tagO* p*tagO*G152A produces 24% WTA relative to the parental RN4220 strain. Wild type flies succumb successively to infection as the levels of WTA increase (log-rank test, P<0.05), likewise for the PGRP-SD mutant. In addition, survival of wild type and PGRP-SD mutant flies increasingly separates for each of the bacterial mutants: wild type versus PGRP-SD, P = 0.2452 (log-rank test, RNΔ*tagO* pMAD); P = 0.0053 (RNΔ*tagO* p*tagO*G152A); P = 0.0001 (RNΔ*tagO* p*tagO*). For all infections, PGRP-SA mutant flies succumb equally to infection (log-rank test, P>0.05).

To further demonstrate the necessity for WTA to protect PG from host recognition, we monitored survival of flies infected with the aforementioned TagO point mutations (Figure 2B and Figure 5). In these experiments, we wanted to rule out unknown causes that may occur due to the absence of the TagO protein *per se*, and also, lessen adverse effects that may occur due to a complete lack of WTA. The survival trend for flies infected with RNΔ*tagO* pMAD, that lacks *tagO* and carries an empty pMAD plasmid vector (vector control), was similar to that for NCTCΔ*tagO*: the PGRP-SA mutant succumbed rapidly, whereas the PGRP-SD mutant and wild type flies generally survived, their curves being statistically inseparable (Figure 5). The injection of the complemented strain (RNΔ*tagO* p*tagO*) resulted in survival outcomes that were characteristic of NCTC8325-4, with PGRP-SD mutant and wild type flies succumbing to the infection, with their curves being statistically separated (Figure 5). Notably, wild type and PGRP-SD mutant flies infected with RNΔ*tagO* p*tagO* ^G152A^ (which produces ∼24% WTA relative to RNΔ*tagO* p*tagO* but produces similar levels of the TagO protein) [29] survived to intermediary levels (Figure 5). Overall, survival of wild type flies decreased as WTA levels increased (with a concomitant decrease in PGRP-SA binding, Figure 2B), and likewise for the PGRP-SD mutant; with the difference between wild type and PGRP-SD mutant survival successively increasing. In contrast, survival of PGRP-SA mutant flies was independent of WTA levels, with flies succumbing strongly for all infections in a statistically inseparable manner (Figure 5). These data confirmed that it was indeed *in vivo* protection of PG by WTA against the consequences of PGRP-SA binding, and furthermore, suggested that a requirement for PGRP-SD gradually became redundant as WTA levels decreased.

It has been reported previously that D-alanylation of WTA is also required for the pathogenicity of *S*. *aureus* [11]; D-alanylation is a process that incorporates D-alanine residues into the glycerol-/ribitol-phosphate backbone of WTA, thereby reducing the negative charge of the polymer [33]. We examined therefore, whether a *S.* aureus mutant that lacks the D-alanylation pathway (RNΔ*dltABCD*) bound mCherry-PGRP-SA equivalently to RNΔ*tagO*. Binding of, mCherry-PGRP-SA to RNΔ*dltABCD* was similar to the binding to the wild type bacteria (Figure S3). This prompted us to assess how RNΔ*dltABCD* affected survival of the wild type, PGRP-SD and PGRP-SA mutant flies. In contrast to RNΔ*tagO*, PGRP-SA mutant flies did not succumb strongly to RNΔ*dltABCD* infection, with 83% surviving at 72 hours post-infection (Figure S4); furthermore, survival was statistically inseparable for the different fly backgrounds (Figure S4). These data demonstrated that D-alanylation is not necessary for WTA to limit the access of PGRP-SA, that neither PGRP-SD nor PGRP-SA were required to control the RNΔ*dltABCD* infection and consequently, the reduced killing effect of RNΔ*dltABCD* had nothing to do with recognition.

## Discussion

The results shown here indicate that in respect to Gram-positive bacteria, where the cell wall is not concealed by outer membrane (e.g. *staphylococci*), pathogen recognition, via recognition of PG, is tightly linked to host survival. Our studies bring forward the notion that one of the strategies used by pathogens to reduce recognition is to restrict accessibility to inflammatory non-self components of the cell wall. Specifically, the results here show that presence of WTA in a range of Gram-positive bacteria impaired PGRP-SA binding. The use of tunicamycin to abolish WTA synthesis dramatically improved receptor recognition of bacteria as well as host survival of flies infected antibiotic treated *S. aureus*. Genetically deleting a major component of the WTA synthesis (TagO) in *S. aureus* also increased PGRP-SA binding leading to increased host survival. It should also be noted that, rPGRP-SA was capable of binding *in vitro* significantly better to WTA-free PG than to WTA-linked PG that were purified from wild type *S. aureus* bacteria, treated with trypsin to remove any attached surface proteins and adjusted to the same concentration of PG (Figure S2). This observation confirmed the results obtained with live bacteria and allowed us to eliminate the notion that deletion of *tagO* gene may influence the amount of protein present at the cell surface and that this change in protein levels was influencing the binding of PGRP-SA. Effectively during the course of this work we have removed WTA from PG by treatment with antibiotic, by deletion of the *tagO* gene and finally we have chemically removed them from PG. In all the cases binding of PGRP-SA to PG has increased.

*S. aureus* produces WTA composed of about 40 ribitol phosphate-repeating units modified with N-acetylglucosamine (GlcNAc) and D-alanine [7]. The latter modification is mediated by the D-Alanine ligase DltA and partially neutralizes the negative charge of the cell surface thus reducing attraction of cationic AMPs [33]. Δ*dltA* mutants are more susceptible to killing by cationic AMPs and neutrophils *in vitro* and have markedly reduced virulence in several animal infection models including Drosophila [11], [34]. In one of these studies [11], Tabuchi and colleagues showed that *S. aureus* producing WTA without D-alanylation were impaired in their ability to kill *Drosophila*. Surprisingly, the Δ*dltA* mutant was more impaired in the ability to kill flies than an independently generated *tagO* mutant [11]; the latter according to the authors had the same pathogenicity as wild type *S. aureus* [11], contrary to our findings.

There is a crucial point to be made in reference to this however, which is at the heart of our experimental design and gives physiological relevance to our results. We propose that WTA are important to reduce *S. aureus* recognition by the host and thus help the pathogen increase its numbers inside the fly. The host uses PGRP-SA to control bacterial numbers and the more PGRP-SA binds to the cell wall (see Figure 2B) the more the bacterial load is controlled (as seen by comparing CFUs between wild type NCTC8325-4 *S. aureus* and NCTCΔ*tagO* in Figure 3A). In PGRP-SA mutants the control mechanism is absent and NCTCΔ*tagO* was able to proliferate and kill the host (Figure 3B). We were able to observe this because we started from a low bacterial load (10^2^ cells/initial infection/fly) and followed the progress of pathogen load inside the host. Tabuchi *et al*. injected 10^4^–10^6^ cells per fly for all bacterial strains used [11]. In our hands this concentration overwhelmed the host from the beginning and it is not surprising that these authors were unable to resolve statistical differences in host survival.

In order to rule out possible pleiotropic effects produced by the inactivation of the *tagO* due to the insertion of non-replicative plasmids or reversion of the mutation by elimination of the plasmid from the chromosome, we have specifically deleted the *tagO* gene in a manner that left no resistance marker in the bacterial chromosome and thus minimized possible alterations on the transcription of neighbouring genes. Finally, in order to increase the confidence of our results, we have complemented the *tagO* null mutant with plasmids that allowed the expression of a partially active (TagO^G152A^), TagO protein and have statistically analyzed the estimated host survival probability curves obtained. Finally we should emphasize that deletion of the *tagO* gene in NCTC8325-4 strain (an agr positive strain) and in RN4220 (an agr negative strains) resulted in similar outcomes (Figure S3) - a reduced pathogenicity in the Drosophila infection model and the production of a bacterial cell surface that was better recognized by mCherry-PGRP-SA.

In parallel experiments we have also generated a Δ*dltA* deletion mutant (this study) as well as a deletion of the Δ*dltA* operon (Δ*dltABCD*) [29] and found that both were indeed less pathogenic than wild type *S. aureus* (Figure S4), similar to what was previously reported [11]. However, this reduced pathogenicity was also observed in PGRP-SA and PGRP-SD single mutant flies (in contrast to Δ*tagO*). This indicated that the non-pathogenicity of Δ*dltA* was not linked to recognition by PGRP-SA or PGRP-SD.

We propose that increased “visibility” of PG to PGRP-SA when WTA were removed, dramatically improved survival of the host. However, alternative interpretations of our results may exist. In the following section we will attempt to challenge and rule them out:

1. We have recently reported that removal of WTA has an impact on PG cross-linking and consequently on the susceptibility to host lysozyme [29]. The possibility that the increased host survival may be the result of decreased pathogen resistance to the lysozyme constitutively expressed in the fly (due to the reduced PG cross-linking in the *S. aureus tagO* null mutant) rather than removal of a physical entity (WTA), which blocked access to PG, was ruled out as follows. We generated an *S. aureus* mutant unable to produce high-level PG cross-linking but capable of producing regular levels of WTA, by deleting the *pbpD* gene [29]. The *pbpD* gene encodes to PBP4 which is responsible for the final stages of PG maturation and results in highly cross-linked PG. As shown in Figure 4, bacteria that produce PG with a low level of cross-linking, but normal levels of WTA, were able to kill wild type flies similarly to the parental *S. aureus* strain. In addition, similar amounts of mCherry-PGRP-SA bound to the surface of both wild type bacteria and NCTCΔ*pbpD* (Figure S3C). These results indicate that in Δ*tagO* increased recognition by mCherry-PGRP-SA and the inability to kill flies is due to the absence of the WTA and not due to modifications in PG cross-linking.
2. The hypothesis that the absence of teichoic acids could turn *S. aureus* bacteria more susceptible to enzymes present in the haemolymph of *Drosophila*, such as lysozyme-like enzymes, which would make the bacteria unable to kill flies, was also considered and ruled out. In accordance with previous reports [34] we have verified that the *S. aureus tagO* null mutant is as resistant to lysozyme as the parental strain. The *tagO* null mutant only becomes susceptible to lysozyme when an additional mutation in the *oat* gene, encoding a protein responsible for PG O-acetylation, is introduced (data not shown). Most importantly, injection of *S. aureus tagO* null mutant into PGRP-SA mutant flies was lethal, indicating that the *S. aureus tagO* null mutant bacteria were able to multiply in the haemolymph of flies if undeterred by PGRP-SA.
3. The possibility that PGRP-SA is responsible for directly killing bacteria lacking WTA was also ruled-out as there was no alteration in the growth rate of *S. aureus tagO* null mutant when grown in the presence of recombinant PGRP-SA (data not shown). PGRP-SA is believed to be non-lytic [35]. Nevertheless, this is a working hypothesis and has not been formally proven. In contrast, an unusual L,D-carboxypeptidase activity has been observed towards PG of some Gram-negative bacteria [36]. At the present moment, we cannot exclude that a protein existing in the haemolymph is capable of mediating killing of *S. aureus tagO* in complex with PGRP-SA. In accordance to the latter hypothesis we have previously shown that PGRP-SA enhances the weak endomuramidase activity of GNBP1 for PG of *M luteus*, the cell wall of which (like *tagO*), is devoid of WTA [37].
4. The possibility that the absence of WTA could turn *S. aureus* bacteria more susceptible to AMPs (produced as a consequence of the recognition of an invading pathogen) was also tested. Injection of *S. aureus tagO* null mutant into mutant flies affected in the ability to produce AMPS, such as *Dif^1^-key* ^1^, *spz^rm7^* and *spz^1^* was not lethal to the flies, indicating that the *S. aureus tagO* null mutant bacteria were being eliminated in a way that was dependent on recognition by PGRP-SA but not dependent upon activation of the production of AMPs (Figure S6). At the moment we are unable to identify how *Drosophila* flies are killing invading *S. aureus tagO* null mutant bacteria. It is possible that bacteria, upon recognition by PGRP-SA, are more easily phagocytised or that, as *in Tenebrio molitor* [38], PGRP-SA binding recruits the local melanization cascade, triggering such a response.

Our results underline an important aspect of pathogen recognition by the host, which remains relatively unexplored. Namely, how does the host recognition machinery respond to changes in the surface of bacteria? Here we manipulated the amount of WTA on the cell surface of *S. aureus*. Previously, two host PGRPs, PGRP-SA and PGRP-SD were found to be involved in recognition of wild type *S. aureus* [14], [15]. We found here that when WTA were genetically removed, the requirement for PGRP-SD was abolished. Flies deficient for PGRP-SD had estimated survival probabilities comparable to wild type flies following infection by *S. aureus* Δ*tagO* or Δ*tagOptagO^D87/D88A^*. When a small amount of WTA was left on the surface through the residual activity of the *S. aureus* Δ*tagOptagO^G152A^* then PGRP-SD mutants were less able to survive infection. However this sensitivity was not as pronounced as when infected with *S. aureus* Δ*tagOptagO*, the strain with reconstituted wild type levels of WTA. Previous studies have established that PGRP-SD does not bind Gram-positive Lys-type PG [14], [39]. However, in its presence, PGRP-SA was able to bind substantially better to cell wall from *S. aureus* and *S. saprophyticus* [14]. Our results, combined with the latter observation, support a role for PGRP-SD in neutralizing the effect of WTA obstructing access to PG. The alternate hypothesis that PGRP-SD may directly recognize WTA, and is therefore not necessary when flies are infected with bacteria that lack teichoic acids, is also a possibility.

The role of teichoic acids in concealing PG at the surface of Gram-positive bacteria may be also effective in preventing recognition by innate immune sensors of other organisms. It is now established that insect PGRPs have mammalian homologues and mice and humans express four genes encoding members of this family [35]. Our results correlate with data, which attributed a significantly reduced virulence of *tagO* mutants in cotton rat nasal colonisation model [40] as well as a mouse endophthalmitis model [41] and suggest a mechanism for how this may happen: absence of teichoic acids may render PG at the bacteria surface more exposed to the host immune system.

## Materials and Methods

### Microbial and fly strains

Isogenic wild type flies (Bloomington #25174) were used as the wild type control. For the survival and bacterial Colony Forming Unit (CFU) experiments, and *DD1* flies for assaying *Drs* levels visually or via qPCR; the latter carries a *Drs-GFP* and a *Diptericin-lacz* reporter [42]. The PGRP-SA and PGRP-SD mutant backgrounds are, respectively: flies with the *semmelweis* mutation in *PGRP-SA* [16] and a 1499 bp deletion in *PGRP-SD* (*PGRP-SD^Δ3^*) [15]. The *spz^rm7^* [43] and *spz^1^* [44] Toll pathway mutant backgrounds, and the *Dif^1^-key^1^* [45] Toll-IMD pathways double mutant background, were used to assess survival of flies deficient for AMPs. All fly stocks were reared at 25°C. Bacterial strains are listed below. *S. aureus* strains were grown in tryptic soy broth medium (TSB; Difco) supplemented with antibiotic (erythromycin 10 µg/ml; Sigma-Aldrich) when required. *E. faecalis* was grown in brain heart infusion medium (BHI; Fluka). *M. luteus* was grown in Luria-Bertani medium (LB; Difco). Bacteria were plated from -80°C stocks every 7 days Growth of all bacteria cultures were done at 30°C as *S. aureus* mutants impaired in the synthesis of teichoic acids are thermosensitive [46].

### *S. aureus* strains

NCTC8325-4 (*S. aureus* reference strain from R. Novick); NCTCΔ*tagO (*NCTC8325-4 *tagO* null mutant [29]); RN4220 (Restriction deficient derivative of *S. aureus* NCTC8325-4 that can be electroporated); RNΔ*tagO* (RN4220 *tagO* null mutant [29]); RNΔ*tagO*pMAD (RNΔ*tagO* transformed with pMAD [29]– shuttle vector with a thermosensitive origin of replication for Gram-positive bacteria); RNΔ*tagO* p*tagO (*RnΔ*tagO* transformed with p*tagO* [29]); RNΔ*tagO* p*tagO* ^D87A/D88A^ (RNΔ*tagO* transformed p*tagO* ^D87A/D88A^ [29]); RNΔ*tagO* p*tagO* ^G152A^; RNΔ*tagO* transformed with p*tagO* ^G152A,^ [29]); RNΔ*dltABCD* (RN4220 *dltABCD* null mutant [29]); RNΔ*dltABCD* (RN4220 *dltABCD* null mutant [29]); RNΔ*dltA* (RN4220 *dltA* null mutant, this study). *M. luteus* strain**:** DMS20030 [47]; *E. faecalis* strain**:** JH2-2 [48]; *B. subtilis* strain MB24 [49].

### Construction of the RNΔ*dltA* null mutant

To delete the *dltA* gene from the chromosome of *S. aureus* RN4220 we started by amplifying two 0.55 Kb DNA fragments from the genome of *S. aureus* NCTC 8325-4 strain, corresponding to the upstream (primers 5′-AGATCTgaatgtatatatttgcgctgatg-3′ and 5′-gtaaaatcaccatatggaatcatattaagtctccctcattagaactc-3′) and downstream (primers 5′- gagttctaatgagggagacttaatatgattccatatggtgattttac-3′ and 5′-GAATTCcgaaacgtttgtaacgatcg-3′) regions of the *dltA* gene. The two fragments were joined by overlap PCR using primers P33 and P36 and the resulting PCR product was digested with *Bgl*II and *Eco*RI and cloned into the pMAD vector, producing the plasmid pΔ*dltA.* This plasmid was sequenced and electroporated into *S. aureus* RN4220 strain. Insertion and excision of pΔ*dltA* into the chromosome of RN4220 was performed as previously described [29] with the exception of the incubation temperature after excision of the plasmid, which was 30°C (instead of 43°C) due to the thermosensitive nature of the cells lacking D-alanylation. Deletion of *dltA* was confirmed by PCR, and the resulting strain was named RNΔ*dltA*.

### Survival experiments and determination of CFUs

Overnight 10 ml cultures of bacteria were washed and resuspended in an equal volume of sterile phosphate buffered saline (PBS), and further diluted 1/1000. Healthy looking adult flies from uncrowded bottles, 2–4 days old, were injected in the thorax with 32 nl of a bacterial cell suspension or PBS using a nanoinjector (Nanoject II, Drummond Scientific). For determination of CFUs, injected flies (6 females) were crushed immediately in media appropriate for the bacteria injected and the homogenates were diluted and plated on tryptic soy agar-media (TSA). The plates were incubated at 30°C for 20–30 hours and the colony forming units (CFUs) per fly were measured by counting the number of colonies on each plate, the CFUs per fly were used to adjust the initial dose of bacteria injected to approximately 100 CFUs per fly. For the time course (0, 6, 17 hours) determination of CFUs, each value represents an arithmetic average derived from three biological repeat experiments (*n* = 3). Flies for survival and PGRP-SA mutant rescue assays were inoculated concurrently with those for determining CFUs, with ten or fifteen flies of each sex injected per bacteria-fly strain combination (or PBS-fly strain); each combination being repeated independently three times (*n* = 3). Following injection, flies were transferred to 30°C and survival assessed every 24 hours over a period of 3 days. Since the trends in survival were the same (i.e. survival curves were positioned similarly relative to one another) for each independent biological repeat, the data for each bacteria-fly strain combination was added (*n* = 60 or *n* = 90) and estimates of survival curves constructed. Flies injected with PBS were mostly unaffected for all fly backgrounds.

### Purification of recombinant rPGRP-SA and mCherry-PGRP-SA from *E.coli*

A truncated version of PGRP-SA (in which the N-terminal sorting sequence was replaced with a T7 tag, and a poly-histidine tag was added to the C-terminus) was expressed in *E. coli* and purified using cobalt affinity resin (Talon; BD Biosciences) under denaturing conditions. A mCherry tagged derivative, mCherry-PGRP-SA was produced using the same procedure. Proteins were stored in 20 mM Tris-HCl pH 8.0 and 150 mM NaCl.

### Protein functionality assays

Functionality assays of the rPGRP-SA and mCherry-PGRP-SA proteins were performed as previously described [14]. *Drs-GFP* expression was monitored after 24 hours of the *M. luteus* infection through the production of fluorescent signal produced by the infected flies; and by qPCR using as template RNA extracted from 6 infected female flies, similar to what was previously described [50].

### Purification of peptidoglycan

Peptidoglycan was prepared from exponentially growing cultures of *S. aureus*, *B. subtilis*, *M. luteus*, and *E. faecalis* as previously described [13].

### PGRP-SA-peptidoglycan co-precipitation assay

50 µg of recombinant PGRP-SA was incubated with 0.2 mg of peptidoglycan and 17 µg of BSA (New England Biolabs) in 20 mM Tris-HCL pH 8.0 and 300 mM NaCl in a final volume of 300 µl. Incubation was at 25°C with agitation for 30 minutes. Peptidoglycan and co-precipitated proteins were harvested by centrifugation, washed twice with 20 mM Tris-HCl pH 8.0, 300 mM NaCl and then resuspended in 1× SDS loading buffer, boiled for 5 minutes and run on 12% SDS PAGE mini gels. An aliquot of the supernatant, representing unbound protein, was also run. Gels were stained with Coommasie stain, destained and imaged using an ImageScanner (Amersham Biosciences/GE Healthcare). Quantifications of bands performed using ImageJ software [51]; each value represents an arithmetic average derived from three biological repeat experiments (*n* = 3).

### mCherry-PGRP-SA binding to bacteria

Bacteria were grown to mid-exponential phase. Washed cell cultures in PBS (500 µl) were incubated with 50 µl of mCherry-PGRP-SA (2 mg/ml in 150 mM NaCl, 20 mM Tris pH 8.0) for 5 minutes on ice. The cells were washed twice with PBS and harvested at 4°C (3000 rpm, 10 minutes). Finally the bacteria were resuspended in 20 µl PBS. A drop of this culture was placed on a PBS, 1% agarose slide and visualised. Images were obtained using a Zeiss Axio ObserverZ1 microscope equipped with a Photometrics CoolSNAP HQ2 camera (Roper Scientific using Metamorph software, Meta Imaging series 7.5) and analyzed using ImageJ software.

### WTA extraction

WTA were extracted by alkaline hydrolysis from overnight cultures were analyzed by native polyacrylamide gel electrophoresis and visualized by combined alcian blue silver staining, as previously described [52]. ImageJ software [51] was used to quantify the percentage of WTA produced by each strain as previously described [29]. The signal intensity of each lane was quantified and normalized against the corresponding value for the wild type (considered as 100%).

### WTA inhibition

Tunicamycin minimum inhibitory concentration (MIC) assays were performed as previously described [28]. Overnight cultures of bacteria were grown in antibiotic free medium or in the presence of a subinhibitory concentration of tunicamycin (0.8 ug/ml for *E. faecalis* – 17× less than the MIC - and 0.4 ug/ml for *S. aureus* and *S. saprophyticus* – 32× less than MIC), that doesn't interfere with the bacterial growth rate. For mCherry-PGRP-SA binding assays, overnight cultures were diluted 1∶100 into fresh medium, with or without tunicamycin at the appropriate concentration, and were grown until mid-exponential phase. For survival experiments, we used *S*. *aureus* overnight culture grown with tunicamycin as above described.

### Data analysis

As nonparametric tests lack statistical power with small samples, when required, data sets with three biological repeats (*n* = 3) were transformed to give a normal distribution (Lilliefors test, *P*>0.05) and then checked for equal variance (Levene's test, *P*>0.05); subsequently, data was analysed using parametric tests.

### Binding assays

Data for the PGRP-SA-peptidoglycan co-precipitation assay was normal with equal variance, thus not transformed; One-way ANOVA was applied to the data. For the mCherry-PGRP-SA binding to bacteria assays data (*n* = 50) was non-normal but with equal variance, therefore nonparametric Kruskal-Wallis test followed by Dunn's multiple comparison was applied.

### CFUs

The complete CFU data set exhibited neither normality nor equal variance, and attempts to rectify this by transforming the data failed. Therefore, the data was separated into 6 groups, which were independently transformed via a Box-Cox transformation (Box-Cox returns a λ number, where a transformed data-point  =  data-point^λ^ – 1/λ) to give a normal distribution with equal variance, and statistical analysis performed as described. Firstly, for each bacterial strain (groups 1–3, graphical representations not shown), Repeated Measures Two-way ANOVA was used to look for differences over time and between the fly backgrounds. However, due to interactions between these two factors, Repeated Measures One-way ANOVA with 95% Tukey's HSD Intervals was used to look for differences over time for each particular bacteria strain and fly background combination (i.e. 9 separate tests, data for each was normally distributed with equal variance). Secondly, at each time point (groups 4–6, Figure 3), Two-Way ANOVA was used to look for differences between the bacterial strains and between the fly backgrounds; where there was an interaction between these two factors, One-way ANOVA with 95% Tukey's HSD Intervals was used to look for differences between the fly backgrounds for a particular bacterial strain.

### Fly survival

Estimated survival curves were constructed from the raw data sets and the Log-rank (Mantel-Cox) test used to determine statistical significance between the curves. For clarity in display, 95% confidence intervals have been omitted from the graphs. All data was plotted and analyzed using GraphPad Prism 5 (GraphPad Software, Inc.) or MATLAB R2009a.

## Supporting Information

Figure S1. **Recombinant PGPR-SA proteins rescue** ***Drs*** **expression in** ***D. melanogaster.*** *D. melanogaster* recombinant PGRP-SA proteins were produced in *E. coli*, except Sf9 rPGRP-SA, which was produced in an insect cell line. Flies carrying a *Drs-GFP* reporter were firstly injected with either 10 ng of a recombinant PGRP-SA (+), 10 ng of the fluorescent mCherry-tag (+), or an equivalent volume of sterile PBS when protein was not injected (PBS); after 2 hours the same flies were infected with *M. luteus*. (A) *Drs-GFP* expression was observed after 24 hours (Drs-GFP), and likewise mCherry fluorescence (mCherry). DD1 flies were used as a wild type control for *Drs-GFP* expression upon infection; all recombinant PGRP-SA proteins rescued *Drs-GFP* expression in the PGRP-SA mutant background, whereas the mCherry-tag or sterile PBS did not. (B) The pooled *Drs* mRNA levels (normalised to the non-immune ribosomal gene *RP49*) from 12 female flies was determined 24 hours post-infection via qPCR. For each fly background, the *Drs* mRNA levels induced by *M. luteus* were expressed as fold-change relative to the PBS injection (comparative CT method). Each column represents the mean value for three independent sets of injection (*n* = 3), and the error bars 95% confidence intervals. One-Way ANOVA and 95% Tukey HSD Intervals were used to analyse the data for PGRP-SA mutant flies: significant differences were not found between flies injected with PBS, *M. luteus*, or with only the recombinant proteins. However, the combination of a recombinant PGRP-SA with *M. luteus* greatly enhanced the levels of *Drs* mRNA (*P*<0.05).
(TIF)

Figure S2. **Substrates used in this study.** (A) Substrates used in this study. A schematic representation of the different substrates used in binding reactions in this study. The surface of live cells is very complex and consists of Peptidoglycan (PG) with attached proteins (purple ovoids), large polymers (such as teichoic acids, red spheres) and other covalent modifications (including O-acetylation, blue triangles). The surface of live cells will also be influenced by the presence of other molecules that are not covalently attached to the PG such as lipoteichoic acids (green spheres) which are anchored in the cell membrane and extra cellular proteins which are not covalently linked to the surface or are anchored in the cell membrane (purple stars and purple shapes in the membrane). It should also be noted that the surfaces of live cells are constantly undergoing remodelling processes and that the PG will be growing and dividing. Cell wall (CW) is produced from live cells by a treatment that subjects the cells to mechanical stress followed by boiling in detergent and treatment with proteases, DNases and RNases. CW consists of PG with covalently attached modifications such as teichoic acids and O-acetylation but free of protein, membrane and nucleic acids. PG is produced from CW by treatment with hydrofluoric acid that removes teichoic acids and O-acetylation, leaving just the naked PG mesh. CW and PG are metabolically inert, the structures should not change with time. (B) PGRP-SA co-precipitation assay in the presence of CW and PG. Binding of PGRP-SA to CW produced from NCTC8325-4 is very low (left panel, lane 4). On the other hand, binding of PGRP-SA to PG produced from NCTC8325-4 is high (right panel, lane 4). The difference between CW and PG is the presence or absence of O-acetyl groups and teichoic acids. Removal of these from CW makes the resulting PG a far better substrate for binding of PGRP-SA. (C) PG type and structure of the repeating unit of teichoic acids found in the strains used in this study. Note that the *S. saphrophyticus* strain used here (ATCC 15305) has a similar or identical teichoic acid composition to S.aureus. Most other strains of *S. saprophyticus* contain a teichoic acid based around a glycerol repeating unit. This glycerol repeating unit is modified by the addition of glucose.
(TIF)

Figure S3. **Absence of WTA, rather than reduced cross-linking or D-alanylation of WTA, enhances PGRP-SA binding to the surface of S. aureus.** (A) Secretion of hemolysins was assayed on TSA blood agar plates to determine the *agr* phenotype of the parental *S. aureus* strains, NCTC8325-4 and RN4220, used in this study. The formation of an inner halo of clearing in the plates is due to the action of the δ-hemolysin, only produced by *agr* positive strains. According to this NCTC8325-4 is an *agr* positive (+) strain while RN4220 is an *agr* negative (-) strain. (B) Growth curves of *S. aureus* wild type and mutants strains in TSB. Overnight cultures were diluted to a starting optical density (OD600) of 0.05, and absorbance measurements were taken every 30 minutes. Shown are representative growth curves of experiments conducted in triplicate; generation times shown as arithmetic averages with standard deviations in the table were calculated during the exponential phase of the growth. NCTCΔ*tagO* and NCTCΔ*pbpD* showed similar generation times to the NCTC8325-4 wild type strain. (C) Exponentially growing cells of NCTC8325-4, NCTCΔ*tagO* and NCTCΔ*pbpD* were incubated with mCherry-PGRP-SA. In addition to lacking WTA, NCTCΔ*tagO* produces a PG with a reduced cross-linking, similar to that seen with NCTCΔ*pbpD*. The fluorescent derivative of PGRP-SA protein was not able to the surface of NCTCΔ*pbpD* bacteria that produces teichoic acids at their surface. Exponential phase cells of RN4220 (a laboratory strain that is *agr* defective), RNΔ*tagO* and RNΔ*dltABCD* were also incubated with the protein. The RNΔ*dltABCD* is a mutant strain whose WTA lacks D-alanine residues. The fluorescent derivative of PGRP-SA protein was not able to the surface of RNΔ*dltABCD* bacteria that produces teichoic acids with no D-alanines at their surface. Grey panels are phase-contrast images of bacterial cells (white scale bar represents 1 µm); black panels mCherry-PGRP-SA binding. Images also show that mcherry-PGRP-SA bound strongly to tagO null mutants constructed in both NCTC8325-4 (*agr* positive) and RN4220 (*agr* negative) strains. (D) Estimated survival curves for wild type flies infected with *S. aureus agr* positive (NCTC8325-4 and NCTCΔ*tagO*) and negative strains (RN4220 and RNΔ*tagO*). Flies were infected with ∼100 bacterial cells and fly survival was assessed every 24 hours over 3 days. *S. aureus* RN4220 strain with *agr* negative phenotype is not affected in the ability to kill drosophila flies.
(TIF)

Figure S4. **PGRP-SA mutant flies survive infection by** ***S. aureus*** **strains defective in the D-alanylation of WTA.** The *dltABCD* operon encode proteins involved in the D-alanylation of WTA. Deletion of *dltA*, or of the *dltABCD* operon, result in bacteria that produce D-Alanine free WTA. With all backgrounds, more than 80% of flies survived infection by RNΔ*dltABCD* or RNΔ*dltA*; all curves being statistically inseparable (log-rank, P>0.05). Survival outcomes with the parental RN4220 strain are similar to those seen with NCTC8325-4.
(TIF)

Figure S5. **Survival dynamics prior to 24** **hours post-infection.** As previously performed, the given fly strains (*n* = 90) were infected with either *S. aureus* NCTC8325-4 or NCTCΔ*tagO* strains, and survival monitored every 6 hours. This revealed that PGRP-SA and GNBP1 mutants succumb almost completely to NCTC8325-4 infection after approximately 18 hours, whereas for NCTCΔ*tagO*, this occurs after 24 hours.
(TIF)

Figure S6. **Flies severely compromised in AMP production are able to survive upon infection with** ***S. aureus*** **lacking WTA.** To assess the contribution of AMPs with regards to determining how flies survive infection with NCTC8325-4 or NCTCΔ*tagO*, flies compromised in their ability to produce AMPs (PGRP-SA, Dif-key, spz*^1^* and spz*^rm7^*) were infected (∼100 cells per fly) and survival recorded every 24 hours over 3 days. For each fly background – except wild type – survival curves were statistically inseparable for flies infected with NCTC8325-4 (log-rank test, P>0.05). Flies affected in the production of AMPs succumbed strongly to infection with wild type bacteria NCTC8325-4 by 72 hours, whereas wild type flies survived up to ∼55%. When infected with NCTCΔ*tagO*, survival curves for each fly background were statistically different from the PGRP-SA mutant flies (log-rank test, P>0.05). PGRP-SA mutant flies succumbed to infection, whereas the rest of the mutants containing functional PGRP-SA but affected in the ability to produced AMPs survived up to more than 60% by 72 hours.
(TIF)
