# Supplementary figures and images for: Wall Teichoic Acids of Staphylococcus aureus Limit Recognition by the Drosophila Peptidoglycan Recognition Protein-SA to Promote Pathogenicity
Source: PLoS Pathog. 2011 Dec 1;7(12):e1002421. doi: 10.1371/journal.ppat.1002421 (PMC3228820; doi:10.1371/journal.ppat.1002421)

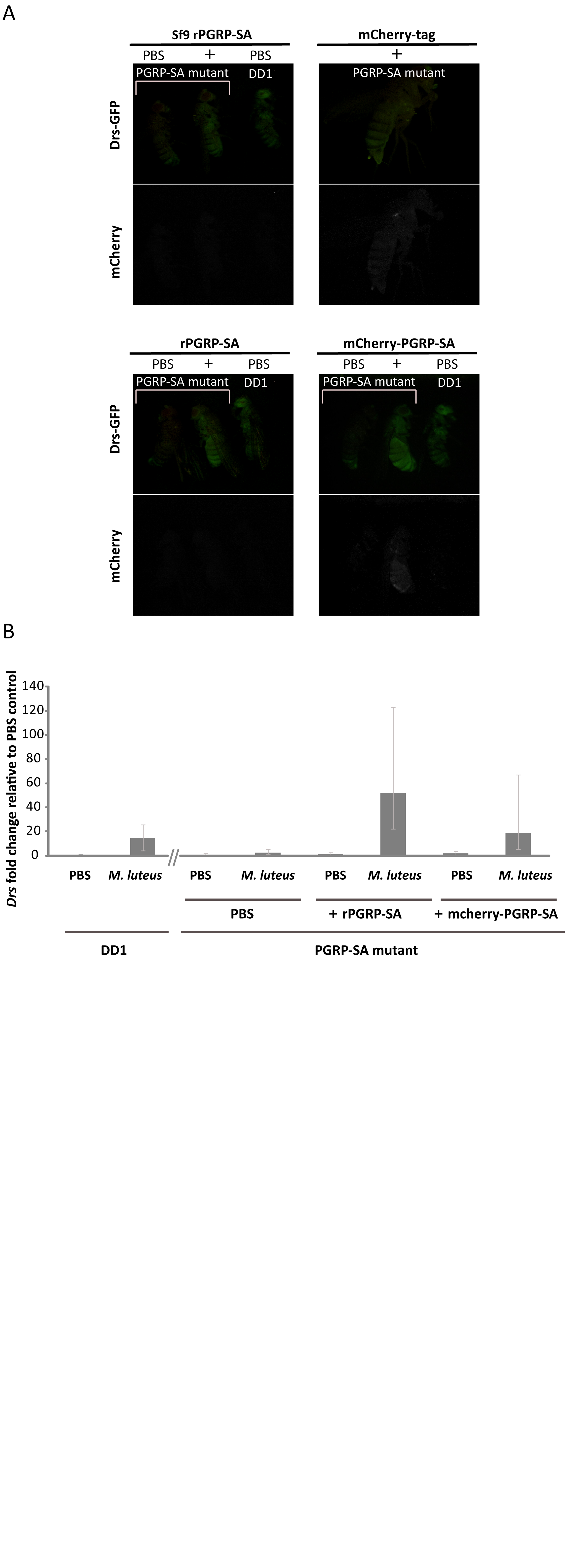

Supplement: Figure S1. — Recombinant PGPR-SA proteins rescue Drs expression in D. melanogaster. D. melanogaster recombinant PGRP-SA proteins were produced in E. coli, except Sf9 rPGRP-SA, which was produced in an insect cell line. Flies carrying a Drs-GFP reporter were firstly injected with either 10 ng of a recombinant PGRP-SA (+), 10 ng of the fluorescent mCherry-tag (+), or an equivalent volume of sterile PBS when protein was not injected (PBS); after 2 hours the same flies were infected with M. luteus. (A) Drs-GFP expression was observed after 24 hours (Drs-GFP), and likewise mCherry fluorescence (mCherry). DD1 flies were used as a wild type control for Drs-GFP expression upon infection; all recombinant PGRP-SA proteins rescued Drs-GFP expression in the PGRP-SA mutant background, whereas the mCherry-tag or sterile PBS did not. (B) The pooled Drs mRNA levels (normalised to the non-immune ribosomal gene RP49) from 12 female flies was determined 24 hours post-infection via qPCR. For each fly background, the Drs mRNA levels induced by M. luteus were expressed as fold-change relative to the PBS injection (comparative CT method). Each column represents the mean value for three independent sets of injection (n = 3), and the error bars 95% confidence intervals. One-Way ANOVA and 95% Tukey HSD Intervals were used to analyse the data for PGRP-SA mutant flies: significant differences were not found between flies injected with PBS, M. luteus, or with only the recombinant proteins. However, the combination of a recombinant PGRP-SA with M. luteus greatly enhanced the levels of Drs mRNA (P<0.05). (TIF) [file ppat.1002421.s001.tif]

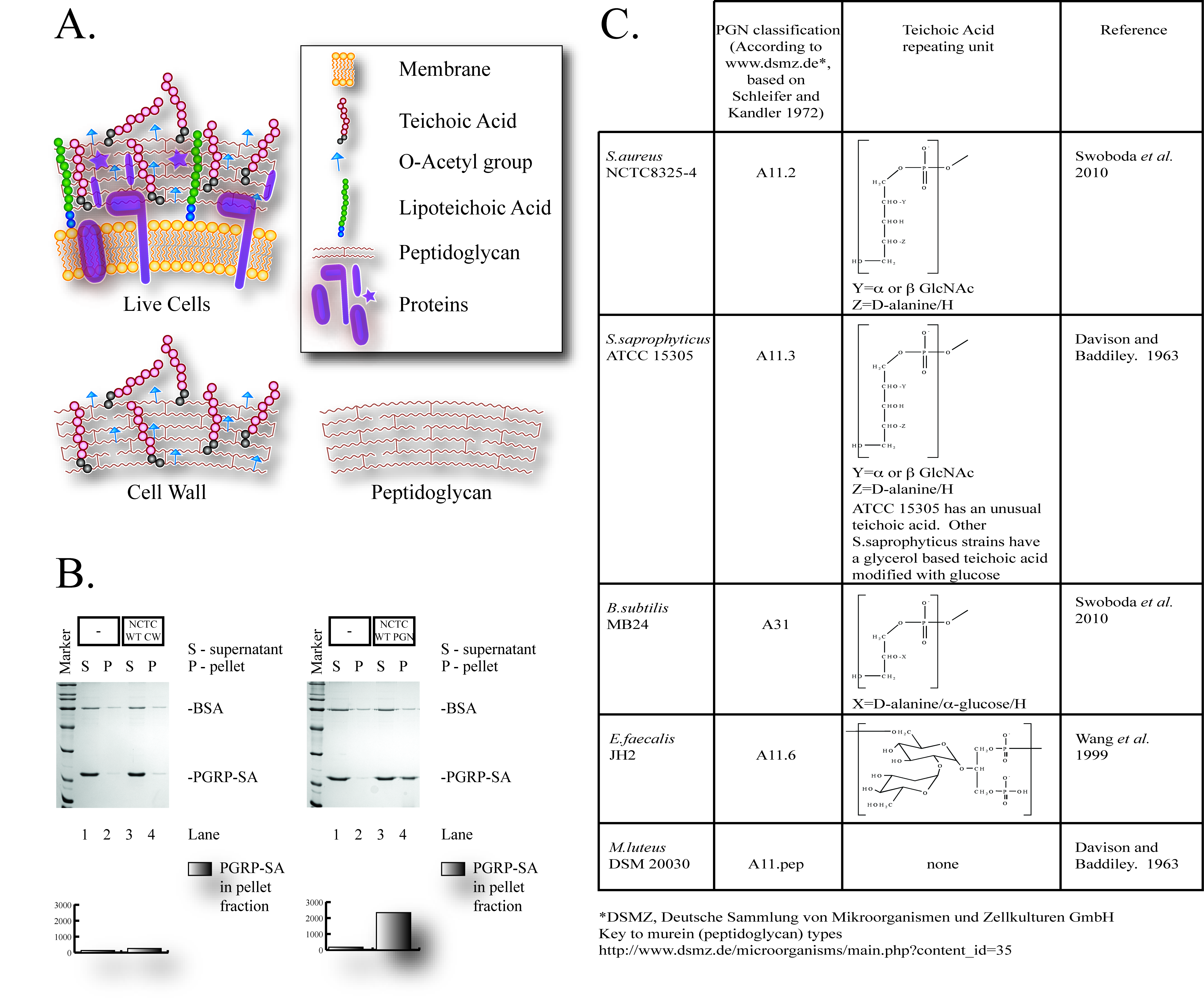

Supplement: Figure S2. — Substrates used in this study. (A) Substrates used in this study. A schematic representation of the different substrates used in binding reactions in this study. The surface of live cells is very complex and consists of Peptidoglycan (PG) with attached proteins (purple ovoids), large polymers (such as teichoic acids, red spheres) and other covalent modifications (including O-acetylation, blue triangles). The surface of live cells will also be influenced by the presence of other molecules that are not covalently attached to the PG such as lipoteichoic acids (green spheres) which are anchored in the cell membrane and extra cellular proteins which are not covalently linked to the surface or are anchored in the cell membrane (purple stars and purple shapes in the membrane). It should also be noted that the surfaces of live cells are constantly undergoing remodelling processes and that the PG will be growing and dividing. Cell wall (CW) is produced from live cells by a treatment that subjects the cells to mechanical stress followed by boiling in detergent and treatment with proteases, DNases and RNases. CW consists of PG with covalently attached modifications such as teichoic acids and O-acetylation but free of protein, membrane and nucleic acids. PG is produced from CW by treatment with hydrofluoric acid that removes teichoic acids and O-acetylation, leaving just the naked PG mesh. CW and PG are metabolically inert, the structures should not change with time. (B) PGRP-SA co-precipitation assay in the presence of CW and PG. Binding of PGRP-SA to CW produced from NCTC8325-4 is very low (left panel, lane 4). On the other hand, binding of PGRP-SA to PG produced from NCTC8325-4 is high (right panel, lane 4). The difference between CW and PG is the presence or absence of O-acetyl groups and teichoic acids. Removal of these from CW makes the resulting PG a far better substrate for binding of PGRP-SA. (C) PG type and structure of the repeating unit of teichoic acids found in th [file ppat.1002421.s002.tif]

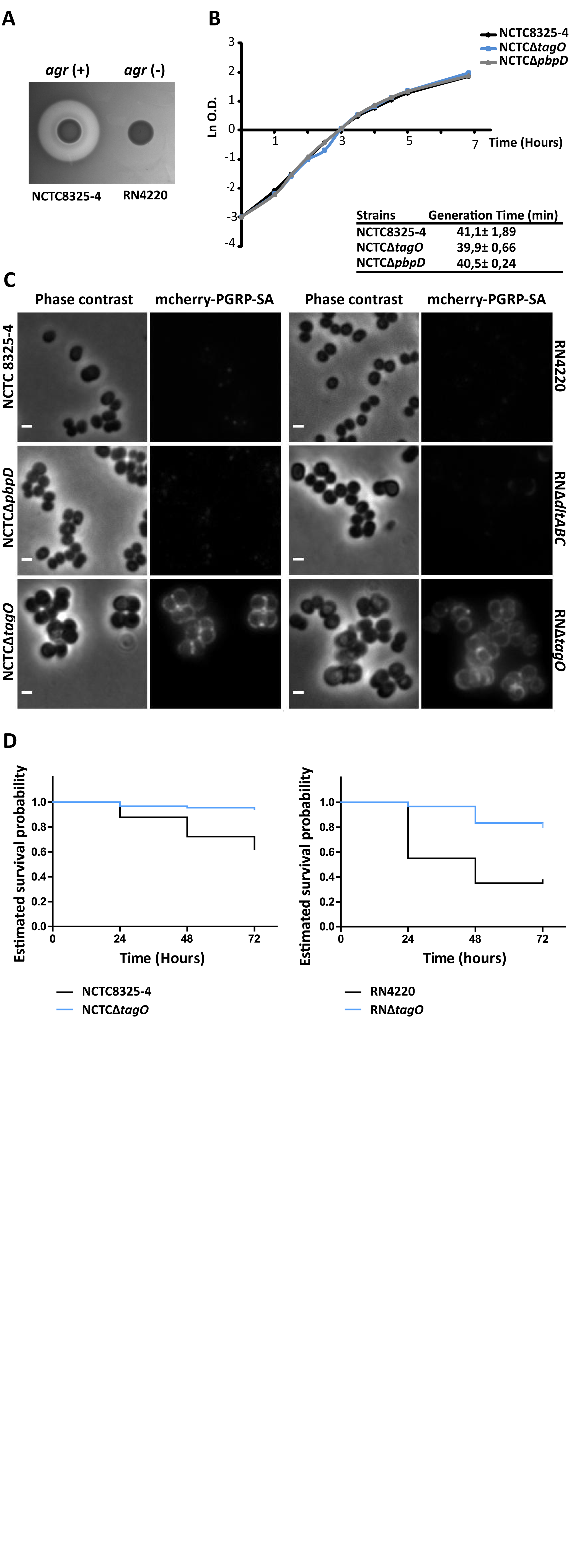

Supplement: Figure S3. — Absence of WTA, rather than reduced cross-linking or D-alanylation of WTA, enhances PGRP-SA binding to the surface of S. aureus. (A) Secretion of hemolysins was assayed on TSA blood agar plates to determine the agr phenotype of the parental S. aureus strains, NCTC8325-4 and RN4220, used in this study. The formation of an inner halo of clearing in the plates is due to the action of the δ-hemolysin, only produced by agr positive strains. According to this NCTC8325-4 is an agr positive (+) strain while RN4220 is an agr negative (-) strain. (B) Growth curves of S. aureus wild type and mutants strains in TSB. Overnight cultures were diluted to a starting optical density (OD600) of 0.05, and absorbance measurements were taken every 30 minutes. Shown are representative growth curves of experiments conducted in triplicate; generation times shown as arithmetic averages with standard deviations in the table were calculated during the exponential phase of the growth. NCTCΔtagO and NCTCΔpbpD showed similar generation times to the NCTC8325-4 wild type strain. (C) Exponentially growing cells of NCTC8325-4, NCTCΔtagO and NCTCΔpbpD were incubated with mCherry-PGRP-SA. In addition to lacking WTA, NCTCΔtagO produces a PG with a reduced cross-linking, similar to that seen with NCTCΔpbpD. The fluorescent derivative of PGRP-SA protein was not able to the surface of NCTCΔpbpD bacteria that produces teichoic acids at their surface. Exponential phase cells of RN4220 (a laboratory strain that is agr defective), RNΔtagO and RNΔdltABCD were also incubated with the protein. The RNΔdltABCD is a mutant strain whose WTA lacks D-alanine residues. The fluorescent derivative of PGRP-SA protein was not able to the surface of RNΔdltABCD bacteria that produces teichoic acids with no D-alanines at their surface. Grey panels are phase-contrast images of bacterial cells (white scale bar represents 1 µm); black panels mCherry-PGRP-SA binding. Images also show that mcherry-PGRP-SA bound strongly to tagO nul [file ppat.1002421.s003.tif]

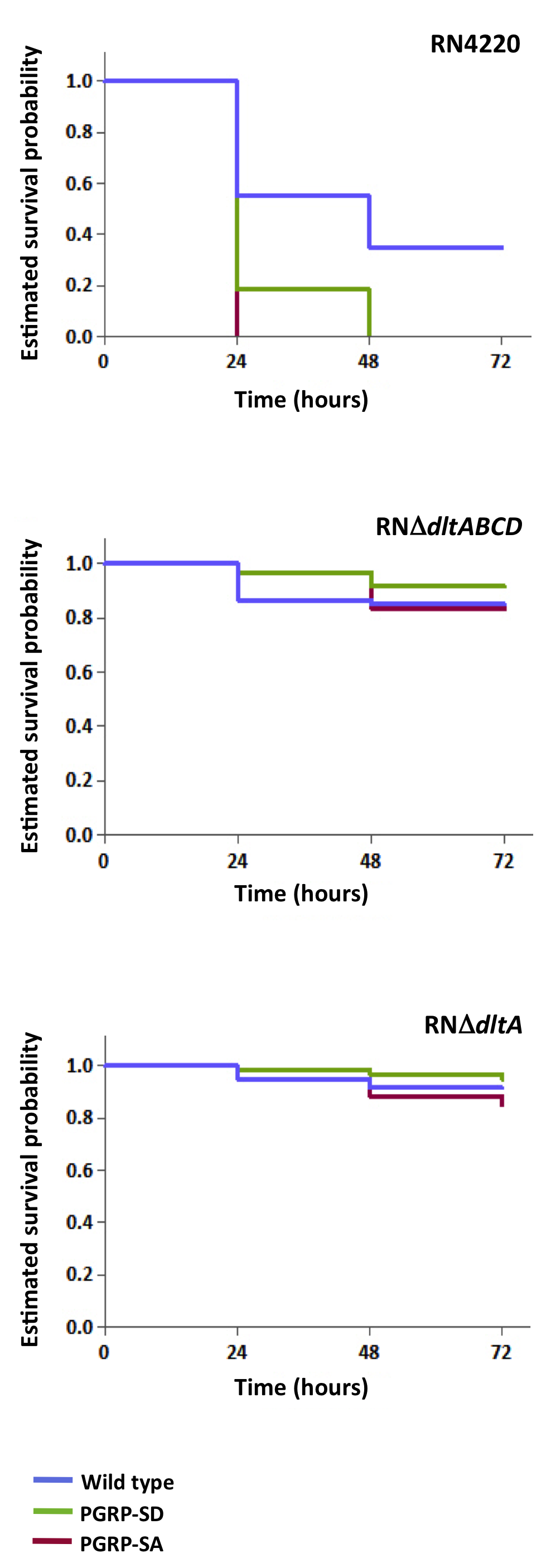

Supplement: Figure S4. — PGRP-SA mutant flies survive infection by S. aureus strains defective in the D-alanylation of WTA. The dltABCD operon encode proteins involved in the D-alanylation of WTA. Deletion of dltA, or of the dltABCD operon, result in bacteria that produce D-Alanine free WTA. With all backgrounds, more than 80% of flies survived infection by RNΔdltABCD or RNΔdltA; all curves being statistically inseparable (log-rank, P>0.05). Survival outcomes with the parental RN4220 strain are similar to those seen with NCTC8325-4. (TIF) [file ppat.1002421.s004.tif]

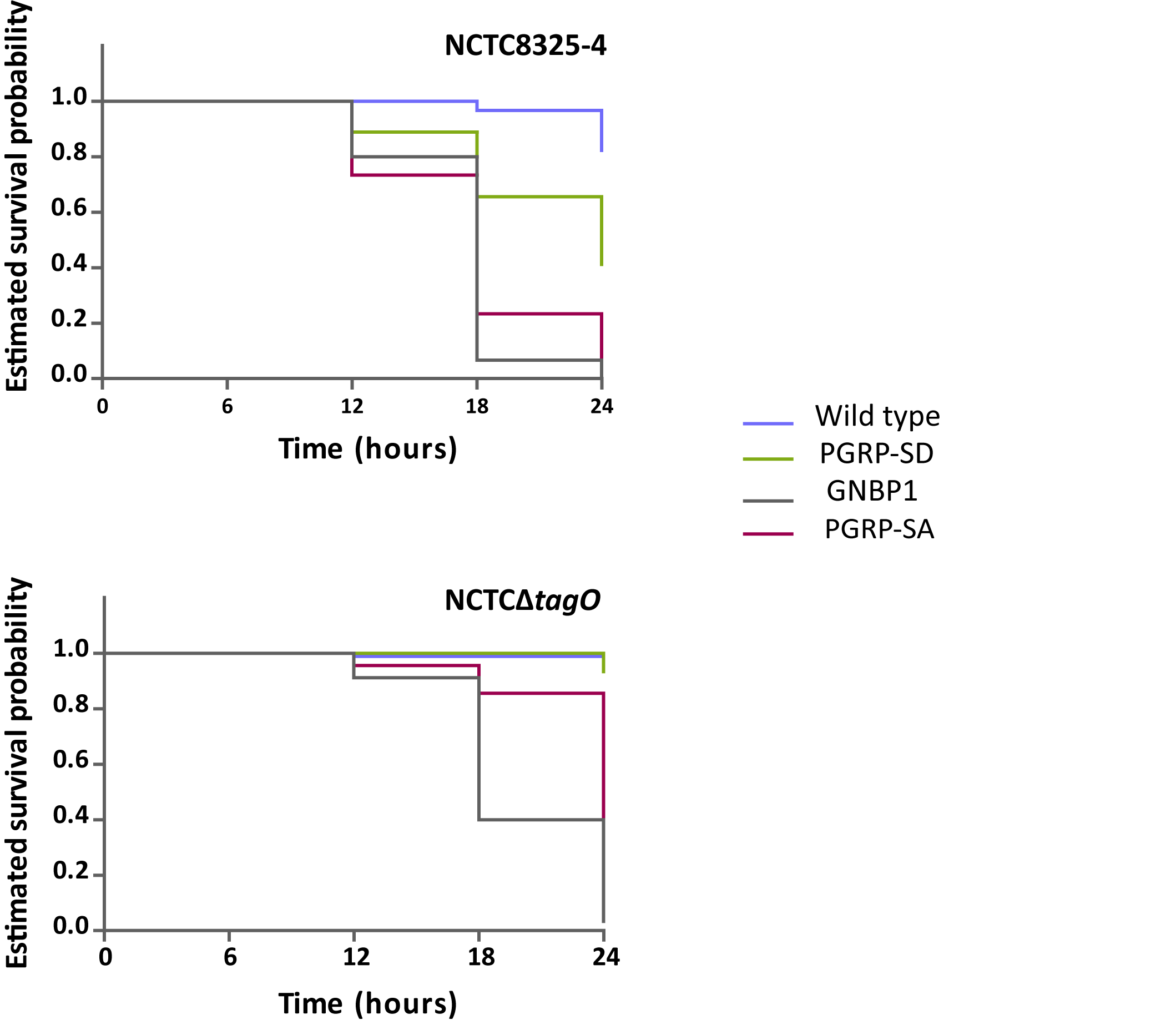

Supplement: Figure S5. — Survival dynamics prior to 24 hours post-infection. As previously performed, the given fly strains (n = 90) were infected with either S. aureus NCTC8325-4 or NCTCΔtagO strains, and survival monitored every 6 hours. This revealed that PGRP-SA and GNBP1 mutants succumb almost completely to NCTC8325-4 infection after approximately 18 hours, whereas for NCTCΔtagO, this occurs after 24 hours. (TIF) [file ppat.1002421.s005.tif]

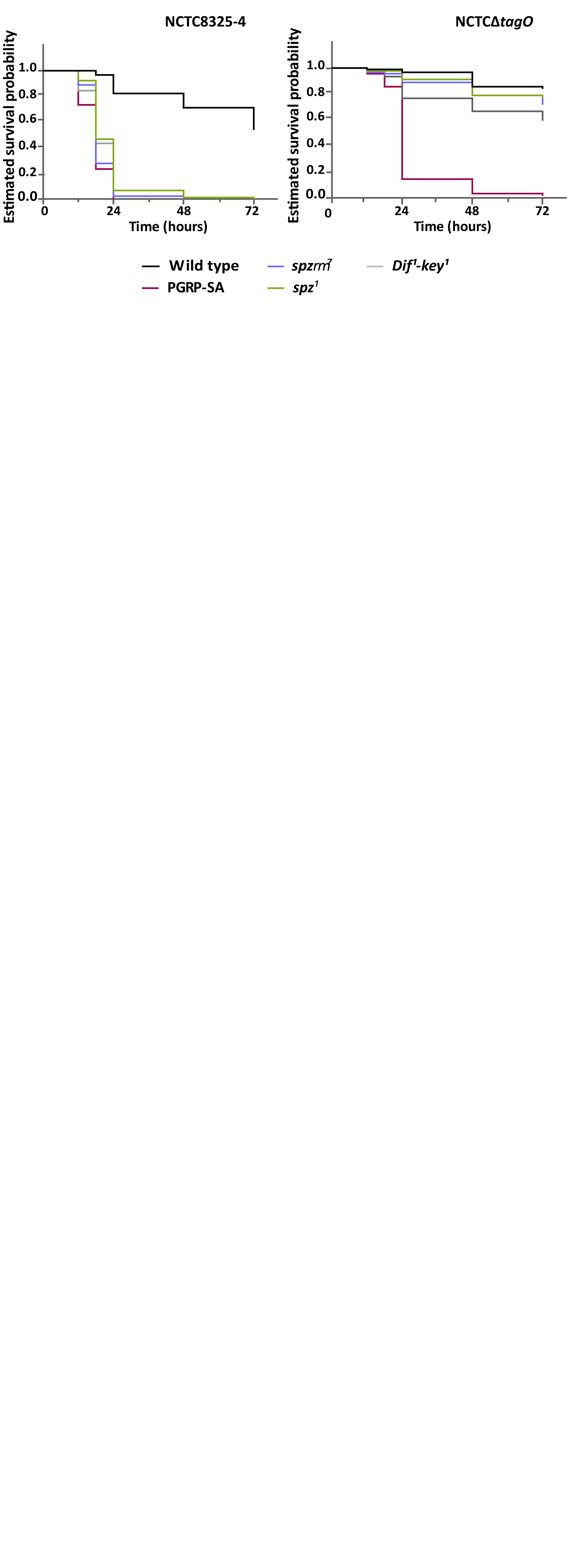

Supplement: Figure S6. — Flies severely compromised in AMP production are able to survive upon infection with S. aureus lacking WTA. To assess the contribution of AMPs with regards to determining how flies survive infection with NCTC8325-4 or NCTCΔtagO, flies compromised in their ability to produce AMPs (PGRP-SA, Dif-key, spz1 and spzrm7) were infected (∼100 cells per fly) and survival recorded every 24 hours over 3 days. For each fly background – except wild type – survival curves were statistically inseparable for flies infected with NCTC8325-4 (log-rank test, P>0.05). Flies affected in the production of AMPs succumbed strongly to infection with wild type bacteria NCTC8325-4 by 72 hours, whereas wild type flies survived up to ∼55%. When infected with NCTCΔtagO, survival curves for each fly background were statistically different from the PGRP-SA mutant flies (log-rank test, P>0.05). PGRP-SA mutant flies succumbed to infection, whereas the rest of the mutants containing functional PGRP-SA but affected in the ability to produced AMPs survived up to more than 60% by 72 hours. (TIF) [file ppat.1002421.s006.tif]
